# Dioxins do not only bind to AHR but also team up with EGFR at the cell-surface: a novel mode of action of toxicological relevance?

**DOI:** 10.17179/excli2024-8038

**Published:** 2025-01-23

**Authors:** Natalie C. Sondermann, Christoph F. A. Vogel, Thomas Haarmann-Stemmann

**Affiliations:** 1 IUF - Leibniz Research Institute for Environmental Medicine, 40225 Düsseldorf, Germany; 2 Department of Environmental Toxicology and Center for Health and the Environment, University of California, Davis, CA 95616, USA

**Keywords:** aryl hydrocarbon receptor, epidermal growth factor receptor, allosteric inhibition, persistent organic pollutants, breast cancer, placental toxicity

## Abstract

Dioxins and dioxin-like compounds (DLCs) are highly toxic organic pollutants whose production and use are prohibited by international law. Despite this, these biopersistent and lipophilic chemicals are prevalent in the environment and accumulate in the food chain, posing significant health risks to consumers even at low exposure levels. Acute dioxin intoxication can cause chloracne, while chronic exposure has been associated with a wide range of adverse health effects, including carcinogenicity, reproductive and developmental disorders, immunotoxicity, and endocrine disruption. In the mid-1970s, scientists identified a transcription factor known as the aryl hydrocarbon receptor (AHR), which becomes activated upon binding of dioxins. AHR orchestrates numerous adaptive and maladaptive stress responses and is believed to mediate most, if not all, of the toxic effects triggered by dioxins and DLCs. Recent studies have provided mounting evidence that dioxins and dioxin-like polychlorinated biphenyls can inhibit growth factor-induced activation of the epidermal growth factor receptor (EGFR) by directly binding to its extracellular domain. This interaction prevents the activation of EGFR by polypeptide growth factors and downstream signal transduction. In this article, we explain this newly identified mechanism of action for dioxins and DLCs in detail and discuss its potential toxicological relevance by using two examples, i.e. breast cancer development and placental toxicity. Finally, we briefly refer to other environmental chemicals of global concern that, based on first published data, may act *via* the same mode of action.

See also the graphical abstract(Fig. 1).

## Introduction

According to the World Health Organization, dietary exposure to 2,3,7,8-tetrachlorodibenzo-*p*-dioxin (TCDD) and dioxin-like compounds (DLCs) remains a significant public health concern (WHO, 2023[103]). These persistent organic pollutants are pervasive in the environment, very lipophilic and tend to accumulate in the food chain. While chloracne is the characteristic symptom of acute intoxication (Furue et al., 2021[19]; Panteleyev and Bickers, 2006[67]), chronic exposure to low doses of dioxins and DLCs can disrupt endocrine functions and impair the immune, reproductive, and developing nervous systems (EFSA Panel on Contaminants in the Food Chain, 2018[15]). Beyond efforts to reduce human exposure, a more comprehensive understanding of the modes of action of dioxins and structurally related chemicals may help to improve risk assessment.

In 1976, Alan Poland and colleagues identified the aryl hydrocarbon receptor (AHR) as a protein that binds radiolabeled TCDD (Poland et al., 1976[73]). Subsequent studies on transgenic mice demonstrated that most of the adverse effects of dioxin exposure depend on AHR (Bunger et al., 2003[6]; Fernandez-Salguero et al., 1996[17]; Mimura et al., 1997[61]; Vorderstrasse et al., 2001[98]), leading to the prevailing view that the toxicity of dioxins and DLCs is exclusively mediated by AHR. However, as discussed below, this picture of a monogamous relationship between dioxins and AHR does not necessarily reflect the true situation. In certain cell types, dioxins interact not only with AHR but also with another key player in cellular signal transduction, i.e. the epidermal growth factor receptor (EGFR). 

In its resting state, AHR is part of a cytosolic multiprotein complex consisting of a heat-shock protein 90 dimer, AHR-interacting protein, co-chaperone p23 and the soluble tyrosine kinase c-Src (Figure 2(Fig. 2)) (Larigot et al., 2022[48]; Vazquez-Rivera et al., 2022[93]). Upon ligand binding, AHR undergoes conformational changes, leading to the dissociation of the multiprotein complex and its translocation into the nucleus, where it dimerizes with the AHR nuclear translocator (ARNT) to form an active transcription factor (Larigot et al., 2022[48]; Vazquez-Rivera et al., 2022[93]). In addition to this canonical AHR pathway, studies from the mid-1980s already showed that TCDD interferes with the activity of EGFR (Astroff et al., 1990[2]; Hudson et al., 1985[28]; Karenlampi et al., 1983[37]; Madhukar et al., 1984[57], 1988[58]), a receptor tyrosine kinase (RTK) integrated in the plasma membrane (Chen et al., 2016[8]), in ways that cannot be explained by AHR activation alone. Specifically, anthropogenic AHR ligands were found to decrease the binding of ^125^I-labeled EGF to the plasma membrane, an observation commonly referred to as EGFR downregulation (Astroff et al., 1990[2]; Hudson et al., 1985[28]; Karenlampi et al., 1983[37]; Madhukar et al., 1984[57], 1988[58]). It was suggested that this might be due to an AHR ligand-enforced internalization of EGFR, an event that follows dimerization, autophosphorylation and activation of downstream signaling molecules (Chen et al., 2016[8]). Unlike polycyclic aromatic hydrocarbons (PAHs), another class of environmental AHR agonists that cause only a transient reduction in EGF binding, TCDD led to a prolonged reduction in EGF-binding capacity, i.e. up to 4 days *in vitro* and 40 days *in vivo* (Hudson et al., 1985[28]; Madhukar et al., 1984[57]). The molecular mechanism underlying this discrepancy remains poorly understood. It has been proposed that AHR ligand-induced internalization of EGFR is due either to its phosphorylation by c-Src (Tice et al., 1999[85]), which is released in the cytosol upon AHR activation (Dong et al., 2011[13]; Kohle et al., 1999[43]; Vogel et al., 2000[94]) or to an enhanced AHR-driven production and release of growth factors that bind to EGFR (Campion et al., 2016[7]; Choi et al., 1991[10]; Du et al., 2005[14]; John et al., 2014[35]; Patel et al., 2006[69]; Sun et al., 2022[82]). However, neither mechanism fully explains the differences in signal transduction observed upon PAH and DLC treatment. 

## A New Mode of Action: Binding of TCDD and Dioxin-Like PCBs to Cell-Surface EGFR

A breakthrough came when Matthew Cave's laboratory for the first time demonstrated that the dioxin-like polychlorinated biphenyl (PCB) congener 126 (as well as non-dioxin-like PCB153) inhibit the growth factor-triggered activation of EGFR, presumably by binding to its extracellular domain (ECD) (Hardesty et al., 2018[24]). Building on this intriguing work, we investigated the crosstalk of AHR and EGFR pathways in response to DLC and PAH exposure in more depth. Our study revealed that EGFR substantially shapes AHR ligand-induced responses in human epithelial cells (Figure 2(Fig. 2)) (Vogeley et al., 2022[97]). Specifically, exposure to the PAH benzo[*a*]pyrene (BaP) as well as to PCB126 resulted in a rapid c-Src-mediated phosphorylation of EGFR within 5-15 minutes after treatment, which confirmed previous reports from TCDD-treated human macrophages and colon cancer cells (Cheon et al., 2007[9]; Xie et al., 2012[105]). In addition, both AHR agonists, BaP and PCB126, stimulated protein kinase C activity and enhanced the ectodomain shedding of cell surface-bound EGFR ligands, namely amphiregulin and transforming growth factor-α (Vogeley et al., 2022[97]). However, only after BaP treatment, this resulted in a timely delayed (2 hours) second peak of EGFR activation and downstream ERK1/2 phosphorylation. Accordingly, hundreds of differentially expressed genes were identified when comparing the transcriptome of BaP- versus PCB126-treated keratinocytes. Subsequent *in silico* docking analyses and EGFR internalization assays confirmed that PCB118, PCB126 and TCDD, but not BaP and benzo[*k*]fluoranthene, bind to the ECD of EGFR and block its activation by polypeptide growth factors (Figure 3A, B(Fig. 3); Reference in Figure 3: Vogeley et al., 2022[97]). Two amino acid residues in close proximity to the binding site for EGF, i.e. Q8 and Q408, were identified to be critical for the binding of dioxins (Vogeley et al., 2022[97]), with the latter residue being also involved in the binding of the EGFR monoclonal antibody cetuximab (Li et al., 2005[49]). The results from the docking simulations are compatible with a model where DLC binding distorts the ECD enough to block EGF binding and subsequent EGFR dimerization. Notably, treatment with PCB126 or TCDD reduced the amphiregulin-induced and EGFR-dependent DNA synthesis in both AHR-proficient and AHR-deficient keratinocytes (Vogeley et al., 2022[97]). An inhibition of the amphiregulin-induced DNA synthesis in AHR-deficient HaCaT cells by the PCB mixture Aroclor 1254 as well as by the non-dioxin-like PCB47, is shown in Figure 2C(Fig. 2). Moreover, the Cave laboratory has reported that treatment of C57Bl/6J mice with Aroclor 1260 resulted in a reduced phosphorylation not only of hepatic EGFR but also of its downstream effectors, such as AKT and mTOR (Hardesty et al., 2017[25]). 

Taken together, these findings may provide a mechanistic explanation for the diverging observations of the mid-1980's studies: Dioxins and DLCs interacted with the EGFR ECD and thereby disturbed the proper binding of (labeled) EGF. Since allosteric inhibition of EGFR may stimulate its subsequent degradation (Perez-Torres et al., 2006[70]; Yao et al., 2020[106]), an additional modulation of EGFR protein levels upon DLC treatment cannot be excluded. Given that ligand-induced AHR activation also results in a transcriptional upregulation and release of EGFR ligands, such as epiregulin, amphiregulin and transforming growth factor-α (Campion et al., 2016[7]; Choi et al., 1991[10]; Du et al., 2005[14]; John et al., 2014[35]; Patel et al., 2006[69]), the extent and duration of this inhibition* via* ECD occupation is probably dose- and time-dependent. Importantly, the described mode of action is still compatible with a rapid endogenous activation of EGFR by dioxins *via* the AHR/c-Src-axis. 

Worth mentioning is that there are reports in literature that are not in line with the proposed mode of action. A study assessing the impact of TCDD on hepatocarcinogenesis in rats, for instance, observed a reduced binding of radiolabeled EGF to hepatic EGFR in the orally-exposed animals (Sewall et al., 1993[76]). Interestingly, the authors could not reproduce this effect in ovariectomized rats, indicating an involvement of estrogen-dependent signaling events (Sewall et al., 1993[76]). However, in contrast to this observation, earlier studies reported a drop in the EGF-binding capacity of hepatic EGFR upon treatment of male rats, hamsters, and guinea pigs with TCDD (Madhukar et al., 1984[57], 1988[58]). 

In addition, multiple other AHR-independent effects of TCDD that do not necessarily involve an interaction with EGFR but possibly with other signaling molecules are described in literature. Examples are the inhibition of migration of AHR-non-responsive human glioblastoma cells (Liu et al., 2024[54]), the induction of endoplasmic reticulum stress in human neuroblastoma cells (Murillo-Gonzalez et al., 2024[65]), and the induction of mitochondrial oxidative stress and insulin resistance in murine myoblasts (Im et al., 2022[29]).

## Allosteric Inhibition of EGFR by DLCs: Clues for Toxicological Relevance?

The novel mode of action of dioxins and DLCs does not only explain ligand-specific differences in the AHR response, but may also account for cell- and tissue-specific effects. EGFR is predominantly expressed in epithelial cells, fibroblasts, and glia cells, and is of fundamental importance for physiology and the development of diseases, including cancer (Chen et al., 2016[8]). Moreover, EGFR-deficient mice die early after birth due to epithelial immaturity and multiorgan failure, demonstrating the critical role of the RTK for proper embryonic development (Miettinen et al., 1995[60]; Sibilia and Wagner, 1995[78]). Hence, the allosteric inhibition of EGFR may be relevant for various diseases and disorders associated with TCDD or DLC exposure, especially those involving dysregulation of cell proliferation, migration, and differentiation. 

The development of chloracne, for example, is linked to dioxin-induced disruptions in the differentiation processes of epidermal keratinocytes and sebocytes (Furue et al., 2021[19]; Panteleyev and Bickers, 2006[67]). *In vitro* experiments have shown that TCDD treatment reduces the proliferation of keratinocytes and accelerates their differentiation (Bhuju et al., 2021[4]; Hudson et al., 1985[28]; Lin et al., 2023[52]; Sutter et al., 2019[84]). The EGFR is an important regulator of keratinocyte function and fate and, accordingly, the switch from proliferation to differentiation can be induced by treating keratinocytes with EGFR inhibitors, too (Joly-Tonetti et al., 2021[36]; Lichtenberger et al., 2013[51]; Peus et al., 1997[71]). In cancer patients, systemic EGFR inhibition is associated with several cutaneous side effects, including aberrant keratinocyte differentiation and skin barrier impairment (Gisondi et al., 2021[21]; Lacouture, 2006[47]). The clinical presentation of EGFR inhibitor-induced skin effects, however, differs from that of dioxin-induced chloracne. While the former are accompanied by inflammatory reactions (Lacouture, 2006[47]), this is usually not the case with chloracne (Furue et al., 2021[19]; Panteleyev and Bickers, 2006[67]). Differences in the underlying pathomechanisms, e.g. EGFR inhibition *versus* EGFR inhibition/AHR activation, may be responsible for this.

However, since chloracne is more relevant in the context of accidental or intentional poisoning with high doses of dioxin, we have decided not to go into further depth but to focus instead on the development of breast cancer and placental toxicity. 

### Development and progression of breast cancer

Depending on cancer type and origin, overexpression or gain-of-function mutations of EGFR may contribute to tumor growth and progression (Uribe et al., 2021[90]). In contrast to the predominating notion that TCDD and related DLCs act *per se* as tumor promoters, several reports also point to anticarcinogenic effects of these environmental contaminants. In fact, two independent studies assessing the chronic toxicity of TCDD in rats in unison reported an increased formation of neoplasms in the lung, liver and oral mucosa, but also a reduced incidence of thyroid, pituitary and mammary tumors (Kociba et al., 1978[42]; Walker et al., 2006[99]). Other studies on rodents revealed that TCDD and DLCs can interfere with tumor growth and progression: Intraperitoneal injection of the PCB mixture Aroclor 1254 reduced the growth of transplanted breast cancer cells in rats (Kerkvliet and Kimeldorf, 1977[40]), TCDD treatment diminished the growth of 7,12-dimethylbenzanthracene-initiated rat mammary tumors (Holcomb and Safe, 1994[27]), and in a mouse model for breast cancer TCDD inhibited metastasis (Wang et al., 2011[100]). Population-based studies found an inverse association between exposure of women to TCDD and PCBs and the risk for hormone-independent breast cancer (Danjou et al., 2015[12]; Gammon et al., 2002[20]). Interestingly, this type of mammary tumor seems to express high levels of EGFR (Masuda et al., 2012[59]). 

In contrast to the anti-carcinogenic effects of TCDD, particularly in the context of breast cancer, multiple epidemiological studies have identified the exposure to other environmentally relevant AHR ligands, i.e. airborne PAHs and PAH-rich particular matter, as a risk factor for breast cancer (Amadou et al., 2021[1]; Mordukhovich et al., 2016[64]; Shen et al., 2017[77]; Smotherman et al., 2023[79]). And indeed, studies have shown that TCDD also exhibits procarcinogenic effects in breast cancer. However, as concluded by a recent systematic review and meta-analysis, the available epidemiological data provide no consistent evidence for an increased risk of breast cancer from TCDD exposure (Cong et al., 2023[11]). One study reporting a positive correlation between dioxin concentrations and breast cancer risk, for instance, is the Seveso Women's Health Study, in which a 10-fold increase in TCDD serum levels was associated with a 2.1-fold increase of the hazard ratio for breast cancer (Warner et al., 2002[101]). Experimental studies on rodents revealed that a maternal exposure to TCDD predisposed the offspring to mammary tumorigenesis (Brown et al., 1998[5]; Jenkins et al., 2007[33]; La Merrill et al., 2010[46]), which might be due to an epigenetic silencing and subsequent downregulation of breast cancer-1 gene expression in the mammary tissue of the maternally exposed offspring (Papoutsis et al., 2015[68]). A study on prostate carcinoma-prone transgenic mice confirmed the procarcinogenic effects of maternal TCDD exposure, while treatment during adulthood significantly delayed the development of neuroendocrine prostate carcinomas in these animals (Moore et al., 2016[63]). A recent study assessing the concentrations of organic pollutants in the adipose tissue of breast cancer patients revealed a positive association between increasing TCDD levels and tumor progression (Koual et al., 2019[45]). With regards to breast cancer progression, activation of AHR, in particular the AHR/c-Src axis (Miret et al., 2022[62]), might play a role (Benoit et al., 2022[3]). In fact, several studies have shown that breast cancer patients with high AHR activity and low expression of the AHR repressor, a negative feedback regulator of AHR (Vogel and Haarmann-Stemmann, 2017[95]), experience shorter metastasis-free survival (Jeschke et al., 2019[34]; Li et al., 2014[50]; Vacher et al., 2018[91]). Along the same line, inhibition of AHR through overexpression of AHR repressor decreased both tumor burden and lung metastasis in the polyoma Middle-T (PyMT) mouse model of breast cancer (Vogel et al., 2021[96]). 

Importantly, EGFR was found to switch its function from proliferative in primary breast tumors to growth-inhibitory in mammary tumor-derived pulmonary metastases (Wendt et al., 2015[102]). Whereas the primary tumor cells responded to Erlotinib treatment, the metastatic cells turned out to be resistant towards pharmacological EGFR inhibition (Wendt et al., 2015[102]). Hence, TCDD may inhibit growth factor-driven EGFR signal transduction in early stages of breast cancer, whereas it is probably ineffective in doing so in advanced stages, when TCDD-activated AHR may dominate and drive tumor progression. However, if an allosteric inhibition of EGFR contributed to the experimentally and epidemiologically observed anticarcinogenic effects of dioxin and DLC exposure is currently not known and urgently requires further investigation. 

### Placental functions and fetal growth

The EGFR is highly expressed in the human placenta and its polypeptide ligand EGF plays a crucial role in regulating placental and fetal growth (Evain-Brion and Alsat, 1994[16]; Fondacci et al., 1994[18]; Rab et al., 2013[74]). Accordingly, altered EGF expression pattern and dysregulated EGFR signal transduction is associated with intrauterine growth restriction (Evain-Brion and Alsat, 1994[16]; Fondacci et al., 1994[18]; Rab et al., 2013[74]), which increases the risk for the development of cardiovascular diseases, type 2 diabetes and other health complications later in life (Knofler et al., 2019[41]). 

Given the high lipophilicity of dioxins and PCBs, these compounds accumulate in the human body and can easily cross the placental barrier and affect the developing life. Experimental studies on rats have shown that *in utero* treatment with TCDD affects the vascular remodeling in the placenta (Ishimura et al., 2006[31], 2009[32]), a process which ensures proper blood supply to the fetus (Knofler et al., 2019[41]). By making use of AHR-null and CYP1A1-null rats as well as different breeding and exposure protocols, Iqbal et al. demonstrated that the effects of *in utero* TCDD exposure on the development of the hemochorial placenta largely depend on maternal AHR signaling (Iqbal et al., 2021[30]). However, a study comparing the effect of TCDD and 2-(1'H-indole-3'-carbonyl)-thiazole-4-carboxylic acid methyl ester (ITE), a potent endogenous AHR ligand (Henry et al., 2006[26]), in pregnant rats, revealed that only TCDD altered the development of the placental vasculature (Wu et al., 2014[104]). Another study comparing the effects of *in utero* TCDD exposure in Holtzman and Sprague-Dawley rats, two rat strains with identical AHR gene sequence, revealed a clearly enhanced susceptibility of the Holtzman strain towards TCDD-induced placental dysfunction and fetal death (Kawakami et al., 2006[39]), again arguing for an additional AHR-independent pathomechanism. Hence, at least in TCDD-exposed rats, AHR-dependent as well as AHR-independent processes may disturb vascular remodeling, an event that may result in undersupply of the fetus and poor fetal growth (Iqbal et al., 2021[30]; Knofler et al., 2019[41]). 

The majority of the available epidemiological studies assessing the impact of maternal DLC exposure on fetal growth indicate an inverse association between DLC serum levels and/or serum AHR activity and birth weight (Govarts et al., 2012[22]; Karmaus and Zhu, 2004[38]; Konishi et al., 2009[44]; Long et al., 2022[55]; Tsukimori et al., 2012[89]; Van Tung et al., 2016[92]; Yen et al., 1994[107]). For example, reduced birth weights correlated with increasing maternal DLC levels in women affected from two mass poisonings in Southeast Asia (Yusho, Japan, 1968 and Yu-Cheng, Taiwan, 1979), during which larger parts of the local population were exposed to PCBs *via *contaminated rice oil (Tsukimori et al., 2012[89]; Yen et al., 1994[107]). Mechanistic studies on placental tissue from mothers four years after being exposed in the Yu-Cheng incident confirmed an intoxication of the tissue with DLCs, which was associated with an elevated expression and activity of CYP1 isoforms (Lucier et al., 1987[56]; Sunahara et al., 1987[83]). However, whereas the capacity of EGFR to autophosphorylate was markedly reduced in the placental tissue of the exposed women, an analysis of the ^125^I-EGF-binding behavior of EGFR did not show any DLC exposure-related differences (Lucier et al., 1987[56]; Sunahara et al., 1987[83]). These findings indicate that the impact of DLC exposure on birth weight is indeed associated with an inhibition of EGFR activity, but this is not necessarily due to a competition between growth factors and DLCs for ECD-binding. However, a study comparing the effect of BaP treatment on the ^125^I-EGF-binding capacity of EGFR in cells isolated from early gestation placentae *versus* cells isolated from term placenta, revealed marked differences (Guyda et al., 1990[23]). Indeed, BaP decreased the capacity of EGFR to bind EGF in the early but not in the term placental cells, suggesting that the inhibitory effect of DLCs on the EGFR ECD might also be of functional relevance only during early placental development. Another process that might contribute to the placental alterations in response to TCDD exposure is the AHR-dependent secretion of IL-24 by chorionic stromal cells, which subsequently inhibited the migration and invasion of placental trophoblasts (Liu et al., 2022[53]), possibly by interfering with growth factor-induced EGFR activation (Poindexter et al., 2010[72]).

However, especially in the light of the novel mode of action discussed in this article, a thorough reassessment of the molecular mechanism responsible for these alterations in placental biology is indicated in order to identify the key event for the developmental toxicity of DLCs. 

## Conclusion

We conclude that in addition to cytosolic AHR, EGFR may serve as a sensor molecule for dioxins and DLCs at the cell-surface. Importantly, this might not only hold true for DLCs but also for other environmental pollutants of global concern, including phenolic benzotriazoles (Sondermann et al., 2024[80]), polybrominated diphenyl ethers (Sondermann et al., 2024[81]), bisphenols (Ticiani et al., 2021[86], 2023[87]) and non-dioxin-like PCBs (Figure 3C(Fig. 3)) (Hardesty et al., 2018[24]; 2017[25]), as well as for the sedative drug phenobarbital (Mutoh et al., 2013[66]). Therefore, exposure to environmental matrices consisting of several of these environmental chemicals can be expected to cause additive effects. However, experimental studies to assess the toxicological relevance of this novel mode of action of organic pollutants have not yet been conducted. Allosteric inhibition of EGFR is an active area of research in oncology (Rybak et al., 2023[75]; To et al., 2022[88]; Yao et al., 2020[106]) and we hope that the arguments summarized in this perspective stimulate further research on this new mode of action in environmental toxicology and will raise the awareness of risk assessors for it. 

## Declaration

### Declaration of competing interest

The authors declare that they have no conflict of interest.

### Acknowledgments

**Figure 1:**
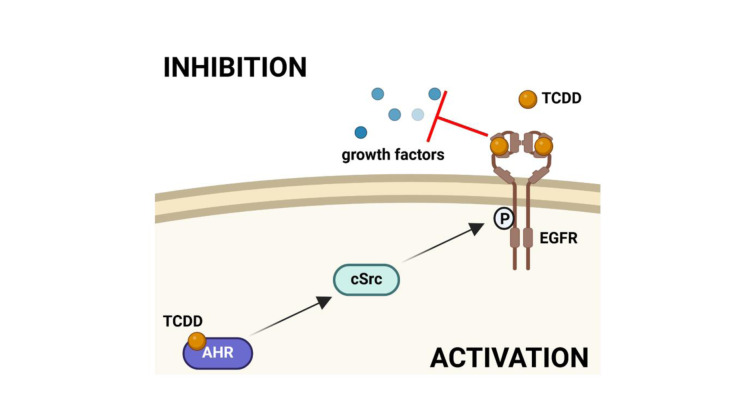
Graphical abstract

**Figure 2:**
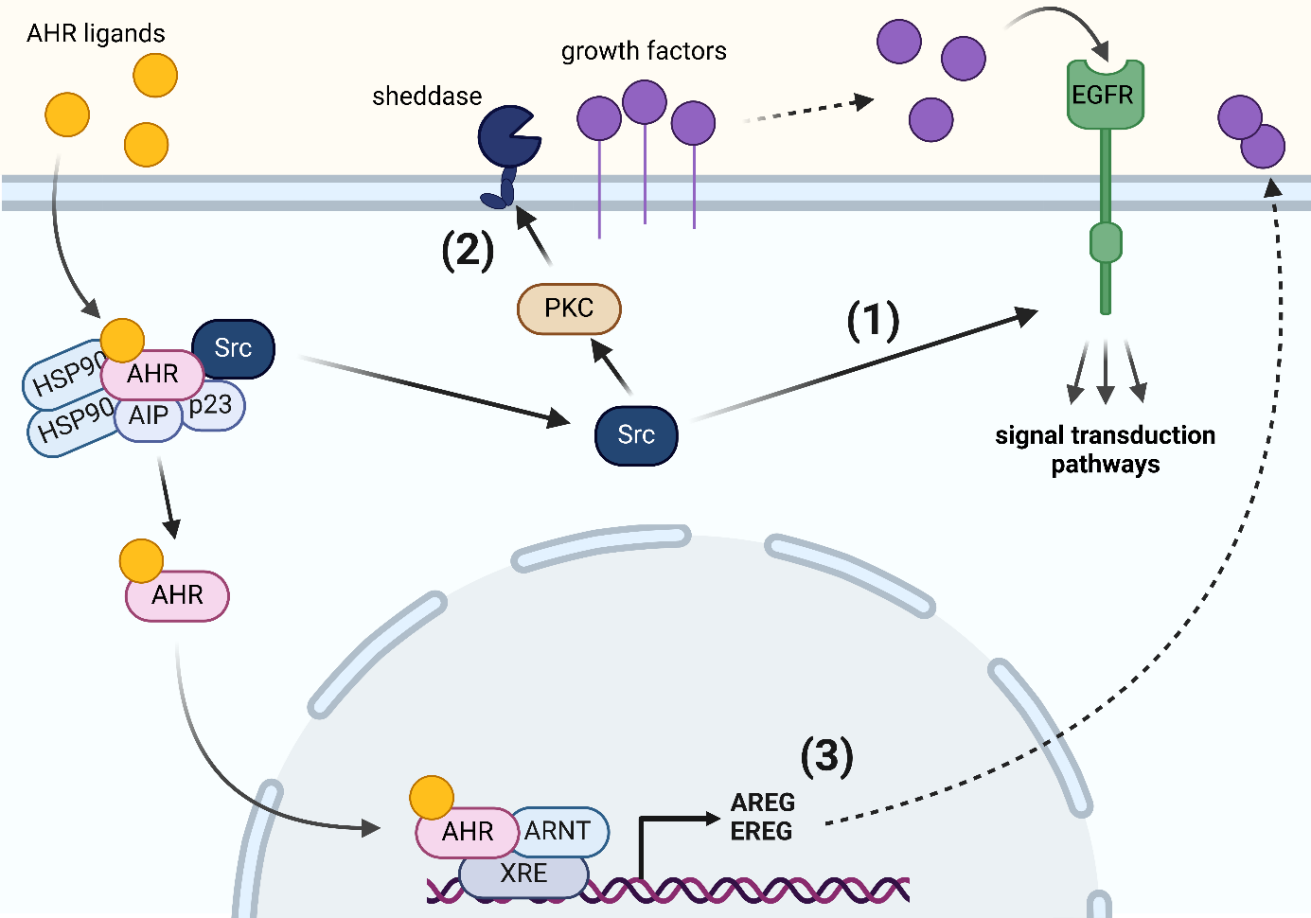
Ligand-induced activation of AHR and its impact on gene expression, EGFR activity and downstream signal transduction. Ligand-binding of AHR leads to the dissociation of the cytosolic multiprotein complex and the nuclear translocation of AHR. In the nucleus AHR dimerizes with ARNT, binds to the enhancer region of genes, for instance encoding the growth factors amphiregulin (AREG) and epiregulin (EREG), and induces their transcription (3). In addition, the ligand-induced dissociation of the cytosolic AHR complex leads to a release of the tyrosine kinase c-Src, which can directly activate EGFR by phosphorylating its intracellular domain (1). Moreover, c-Src sequentially stimulates protein kinase C (PKC) and sheddases, resulting in ectodomain-shedding of cell surface-bound EGFR ligands, such as AREG (2). The released growth factors accumulate and can activate EGFR and downstream signal transduction cascades (e.g. MAPK, PI3K-AKT) by binding to its extracellular domain.

**Figure 3:**
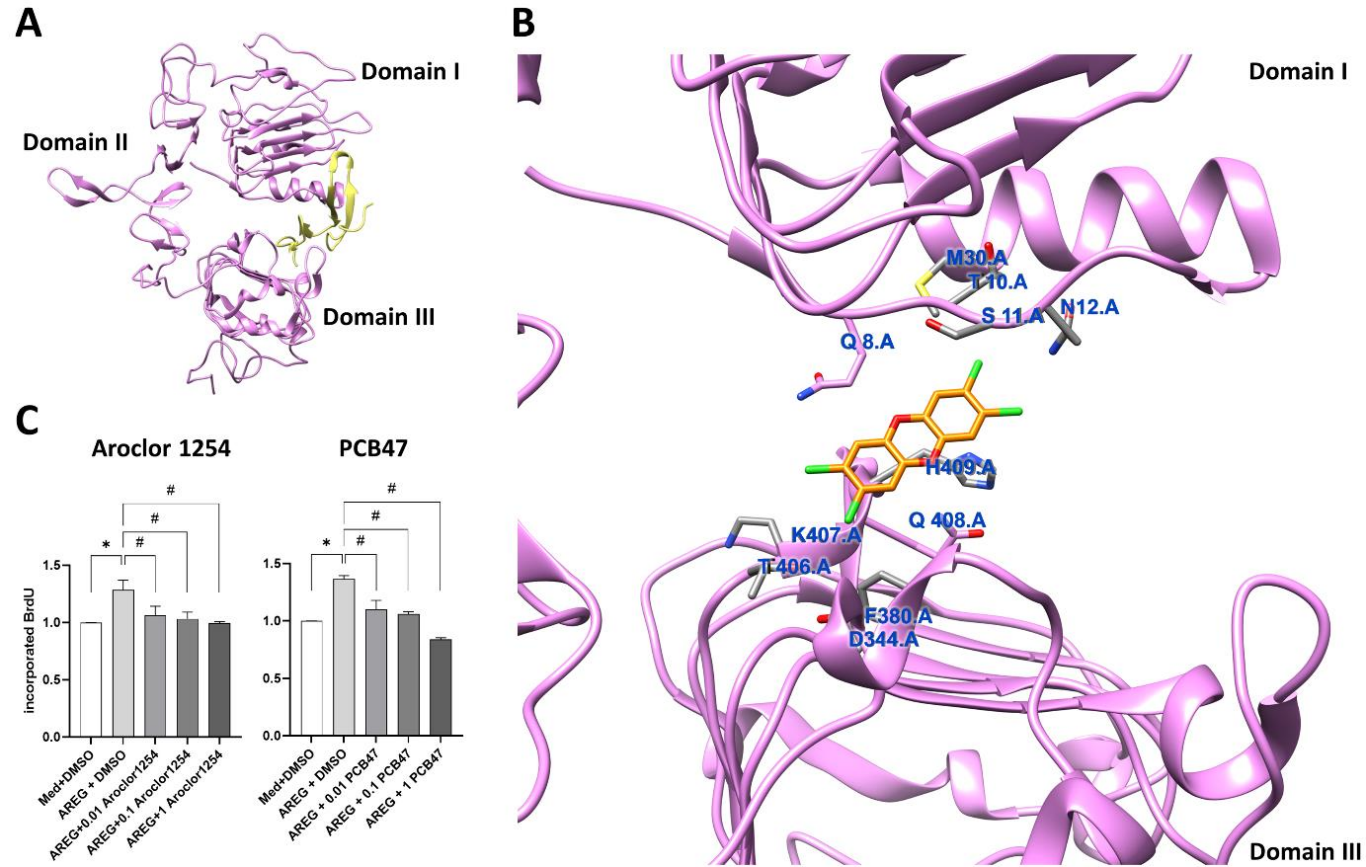
*In silico* docking analysis predicts the binding of TCDD to the EGFR extracellular domain and PCBs inhibit growth factor-induced DNA synthesis in AHR-mutant HaCaT keratinocytes. A. *In silico* docking analysis predicting the binding of EGF (yellow) to the EGFR extracellular domain (magenta). B. *In silico* docking analysis predicting the binding of TCDD to the EGFR extracellular domain in close proximity to the EGF binding site. C. Colorimetric BrdU incorporation assay to assess the influence of the PCB mixture Aroclor 1254 (0.01, 0.1, 1 µM) and non-dioxin-like PCB47 (0.01, 0.1, 1 µM) on DNA synthesis induced by 10 ng/ml amphiregulin (AREG). HaCaT-AHR-mut (DU26) keratinocytes were treated as indicated for 4 h. Absorption was measured at a wavelength of 370 nm (reference wavelength 492 nm). n = 3. *, *p* ≤ 0.05 compared to DMSO. #, *p* ≤ 0.05 compared to AREG/DMSO. For a detailed description of the *in silico* docking analyses, the generation and characterization of the HaCaT-AHR-mut (DU26) keratinocytes, and the BrdU incorporation assay please see Vogeley et al. (2022).

Research in the laboratory of THS is funded by the German Research Foundation (DFG), projects HA 7346/5-1 and HA 7346/6-1. NCS was supported by the Jürgen Manchot Foundation. The National Institute of Environmental Health Sciences (NIEHS)-funded UC Davis EHSC under P30 ES023513, NIEHS R01ES032827 and R01ES036338 supported research in the laboratory of CFAV. The graphical abstract (Figure 1(Fig. 1)) and Figure 2(Fig. 2) were created with BioRender software (www.biorender.com; agreement number TI27NQ6484 and FT27KQ3M1R).
